# Work engagement and associated factors of nurses: a structural equation model

**DOI:** 10.3389/fpsyg.2026.1831111

**Published:** 2026-05-29

**Authors:** Xinmin Zhang, Dan Zhong, Cuicui Li, Yongai Zhang, Miaomiao Zhang, Jintian Zhong

**Affiliations:** 1 Pediatric Orthopedic Hospital, Honghui Hospital, Xi‘an Jiaotong University, Xi'an, Shaanxi, China; 2 The Faculty of Nursing and Rehabilitation, Xi'an Medical University, Xi'an, Shaanxi, China

**Keywords:** leisure crafting, nurses, self-efficacy, thriving at work, work engagement

## Abstract

**Objective:**

To assess the associated factors of work engagement among nurses in tertiary hospitals.

**Background:**

In recent years, the demands for nursing professional skills and quality have gradually increased. Nurses are required to master continuously updated medical knowledge while facing high-intensity work, which may lead to occupational burnout and turnover. Work engagement reflects nursing quality and work enthusiasm and is an important predictor of nurse turnover. Therefore, understanding its current status and associated factors is crucial.

**Methods:**

Using convenience sampling, licensed nurses were selected from Xi'an Honghui Hospital in Shaanxi Province, China. A general information questionnaire and structured questionnaires were used to collect data on demographics, thriving at work, work engagement, self-efficacy, and leisure crafting. A model was constructed to test the research hypotheses. Descriptive analysis was conducted using SPSS 26.0, while AMOS 24.0 was utilized for the verification and analysis of structural equation modeling.

**Results:**

A total of 1,055 questionnaires were distributed, with 1,007 valid responses collected, yielding an effective response rate of 95.45%. The final model explained 80.5% of the total variance in nurses‘ work engagement. Nurses' self-efficacy (B = 0.574, *P* < 0.001), leisure crafting (B = 0.127, *P* < 0.001), and thriving at work (B = 1.157, *P* < 0.001) significantly influenced work engagement. The total effects of self-efficacy and leisure crafting on work engagement were (0.551 and 0.330), respectively; their direct effects were (0.320 and 0.093), and their indirect effects were (0.231 and 0.237).

**Conclusion:**

Chinese nurses exhibit a moderately high level of work engagement, influenced by multiple factors, with thriving at work having the greatest impact.

**Implications for nursing management:**

Nursing managers should prioritize enhancing nurses‘ self-efficacy, encourage reasonable leisure crafting to balance work and life and alleviate work stress, and pay attention to nurses' thriving at work by providing good career development opportunities. These measures can effectively improve nurses' work engagement levels.

## 1 Introduction

Nurses are a key component of the healthcare system and one of the main forces in maintaining the goals of health equity and sustainable health development. In the current stage of rapid development of the medical system, people expect higher quality nursing services and technology. Nurses are required to possess more advanced nursing skills and professional qualities. The “Healthy China 2030” blueprint proposes building a high-level nursing workforce with professional expertise and technical competence, improving educational qualifications, developing specialized nursing, and emphasizing the concept of lifelong education (CPC Central Committee and The State Council, 2016). Nurses need to continuously improve their professional skills throughout their career development to meet societal demands. Therefore, nurses currently face more challenges, such as the high-intensity and high-risk nature of their work, as well as constantly evolving medical knowledge and technologies. These factors result in a heavy workload and significant work-related stress, which to some extent affect nurses‘ career growth and the enhancement of their professional competencies (Van et al., 2019), leading to high turnover and high mobility rates within the industry (Galanis et al., 2025). Research shows that factors such as limited career development, insufficient promotion opportunities, poor working environments, and job burnout significantly increase the risk of nurse turnover (Van et al., 2019; Galanis et al., 2025). Work engagement refers to an individual's multidimensional involvement in their work—physiologically, cognitively, and emotionally—and reflects employees' work enthusiasm (Liu et al., 2025). Multiple studies have demonstrated a significant negative correlation between nurses‘ work engagement and their turnover intention (Lin et al., 2025; Fu et al., 2025; Cao and Chen, 2021). Work engagement not only directly reduces turnover intention but also indirectly influences it by enhancing professional identity, well-being, psychological capital, and other factors (Lin et al., 2025; Fu et al., 2025; Cao and Chen, 2021). At the same time, work engagement can enhance innovative behavior, learning ability, and team collaboration. Highly engaged employees are more motivated to learn proactively and take on challenging tasks (Lin et al., 2025). Currently, there are significant differences in nurse work engagement levels worldwide, with overall room for improvement. Studies from the United States (Wei et al., 2023) and China (Liu et al., 2025) show that nurses' work engagement scores are 3.68 ± 1.05 and 3.3 (2.1, 4.5), respectively, indicating a moderate level. As an important predictor of nurses‘ intention to leave, work engagement is directly related to nurses' work enthusiasm, degree of professional burnout, and the stability of the nursing workforce (Fu et al., 2025; Cao and Chen, 2021). Therefore, gaining an in-depth understanding of the current status of nurses‘ work engagement and its related factors is of great significance for promoting nurses' personal career development and improving the quality of nursing services.

In recent years, scholars have conducted related research on nurses' work engagement. Zhang et al. (2026) surveyed 292 nursing staff and found that change fatigue plays a partial mediating role between perceived organizational support and work engagement, with the indirect effect accounting for 37.79% of the total effect. Li et al. (2026) conducted a latent profile analysis of 542 outpatient nurses and identified three categories of nurse work engagement: “low engagement-burnout type,” “medium engagement-vitality type,” and “high engagement-dedication type,” with different influencing factors for each category. The study by Qing and Luo (2025) showed that the work engagement score of 1,440 clinical nurses from 10 tertiary public hospitals in Sichuan Province was (31.99 ± 6.34), and that nurse-patient relationships and sense of professional mission could explain 44.4% of the variance in work engagement.

However, the aforementioned studies mainly focus on the relationship between a single or a few variables and work engagement (Zhang et al., 2026; Li et al., 2026), or use traditional regression analysis to explore influencing factors (Qing and Luo, 2025), lacking an integrated model constructed based on a systems theory framework, which makes it difficult to reveal the relationships and pathways among various factors. Secondly, some model-based studies on nurse work engagement have been conducted abroad, but differences in cultural background, healthcare systems, and nursing management models limit the direct applicability of their conclusions to China. Based on this, the present study uses the Conservation of Resources (COR) theory as its theoretical foundation to pre-construct a structural equation model including leisure crafting, self-efficacy, thriving at work and work engagement. Through structural equation modeling, a comprehensive statistical method that simultaneously estimates latent variables and parameters of complex predictor/outcome models, the study analyzes the complex relationships among variables in the model, systematically integrates key factors influencing nurse work engagement, reveals their pathways of effect, expands the application scope of COR theory, and provides empirical evidence for hospital managers to identify intervention targets and formulate strategies to enhance nurse work engagement.

### 1.1 Teoretical underpinning

This study is theoretically supported by the Conservation of Resources Theory (COR Theory). The core concept of this theory posits that individuals have an inherent tendency to actively protect their own resources and avoid resource loss. When resources are threatened with loss or when invested resources do not yield the expected returns, psychological stress is triggered, which in turn affects attitudes and behaviors (Chen et al., 2015). Resources refer to anything that individuals perceive as valuable and that can help them achieve goals or cope with stress, including objective resources, condition resources, personal traits, and energy resources (Chen et al., 2015). When a spiral loss of resources occurs, an individual's coping ability may decline, leading to a vicious cycle of further losses. Conversely, when resources are gained, it enhances the individual's coping ability, making it easier to acquire new resources and forming a virtuous cycle (Hobfoll, 2001).

This theory provides a framework for understanding nurses' work engagement. Nurses' work engagement is actually a complex psychological and behavioral process influenced by multiple dimensions and factors, closely related to personal traits, objective resources, conditional resources, and energy resources (Prapanjaroensin et al., 2017). Objective resources include income and work environment; conditional resources encompass marital status, professional status, level of career development, and the ability to shape leisure time; personal traits include self-efficacy; and energy resources cover time, experience, and health. All of these factors influence an individual's attitudes and behaviors. Structural equation modeling, as a powerful statistical analysis tool, can simultaneously handle relationships among multiple dependent and independent variables and reveal their direct and indirect effects. It provides a statistical method for in-depth exploration of the factors influencing nurses' work engagement and the mechanisms of their interactions.

Thriving at work refers to individuals achieving personal growth by feeling energized and continuously acquiring and applying knowledge (Kleine et al., 2019). Thriving at work is defined as the combined experience of vitality and learning, where vitality, as a positive affective experience, represents an individual's well-being; learning, as a pathway to growth, reflects proactive behavioral intentions (Spreitzer et al., 2005). Numerous studies have shown that thriving at work is positively correlated with work engagement, organizational commitment, and innovative work behavior (Liang et al., 2024; Zhai et al., 2023). It can effectively reduce job burnout, stress, and turnover intentions while enhancing job satisfaction (Liang et al., 2024; Zhai et al., 2023). In high-stress industries such as nursing, thriving at work significantly improves team performance and employee adaptability (Liang et al., 2024). Based on the above perspectives, it is reasonable to hypothesize that nurses' thriving at work is positively correlated with work engagement.

H1: Thriving at work is positively correlated with work engagement.

Self-efficacy refers to an individual's belief and confidence in their ability to successfully complete a specific task or handle a particular challenge (Fan et al., 2026). High self-efficacy not only boosts nurses' confidence but also enhances their adaptability to stress and uncertainty, promoting positive work experiences and well-being. The higher the nurses' self-efficacy, the greater their levels of thriving at work and work engagement. Multiple studies have consistently found that nurses with higher self-efficacy are better able to actively cope with work challenges, demonstrating greater focus, dedication, and professional vitality, thereby fostering higher thriving at work and work engagement (Cabrera-Aguilar et al., 2023; Al-Hamdan and Bani Issa, 2022; Morales-García et al., 2024). Therefore, it is reasonable to hypothesize that nurses' self-efficacy is positively correlated with thriving at work and work engagement.

H2: Self-efficacy is positively correlated with thriving at work.

H3: Self-efficacy is positively correlated with work engagement.

Leisure crafting refers to an individual's proactive engagement in goal setting, socializing, learning, and self-development activities during their leisure time (Guo et al., 2022). Research has shown that nurses‘ leisure crafting is significantly positively correlated with work engagement (Liu et al., 2024). When nurses actively plan and utilize their free time through meaningful leisure activities to replenish resources and restore energy, it has been proven to significantly promote their professional growth and job well-being. The higher the level of leisure crafting, the greater the nurses' work enthusiasm, innovation ability, and professional commitment (Liu et al., 2024). Leisure crafting not only meets nurses‘ needs for personal growth and career development but also stimulates positive professional attitudes, drives reflective practice, and broadens professional perspectives (Wu et al., 2025). Therefore, it is reasonable to predict a positive correlation between nurses' leisure crafting and both thriving at work and work engagement.

H4: Leisure crafting is positively correlated with thriving at work.

H5: Leisure crafting is positively correlated with work engagement.

**Figure 1:**
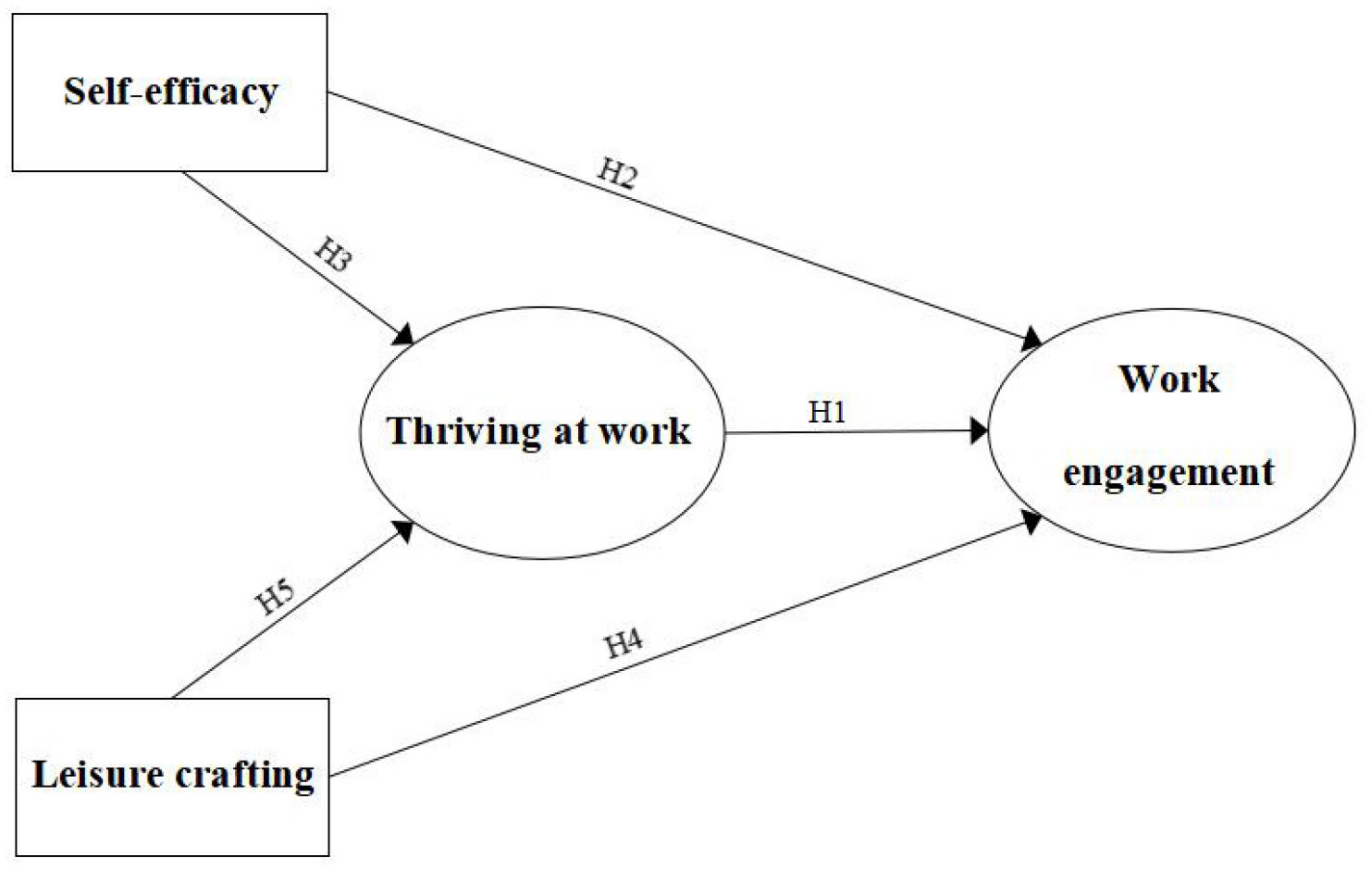
Theoretical framework.

Based on the Conservation of Resources theory and previous literature reviews, this study proposes a theoretical framework with work engagement as the dependent variable, thriving at work as the mediating variable, and self-efficacy and leisure crafting as the independent variables, as shown in Figure 1.

## 2 Methods

### 2.1 Research subjects

Using convenience sampling, clinical nurses from Xi‘an Honghui Hospital were selected as research subjects during the period from January to February 2025. Xi'an Honghui Hospital is one of the large tertiary hospitals in Northwest China. At the time of the study, the hospital had a total of 2,828 beds and 1,526 registered nurses on staff, ensuring that this study could obtain a sufficient number of valid samples.

The project leader contacted members of the hospital's various departmental research teams to introduce the purpose, significance, and data collection methods of this study, and obtained their consent to serve as investigators for this research. These investigators were trained in the implementation of the study according to a standardized code of conduct for research surveyors. Subsequently, the investigators organized the questionnaire collection within each department, explained the study's purpose, significance, basic questionnaire content, and instructions for completion to the participants. After obtaining informed consent, electronic structured questionnaires were distributed via the online platform “Wenjuanxing” to eligible participants who met the inclusion criteria.

Inclusion criteria: Possession of a nurse practitioner qualification certificate and completed registration; Employed for at least 3 months; Provided informed consent and voluntarily participated in the study. Exclusion criteria: Interns, nurses in standardized training, or nurses on advanced study; Nurses absent from work during the survey period due to reasons such as sick leave or maternity leave; Questionnaires submitted in less than 2 min or more than 15 min; Responses showing identical answers across consecutive items, patterned answering, or contradictory answers on reverse-coded items.

According to the sample size calculation method of structural equation modeling, which recommends 15–20 times the number of observed variables (Hoyle and Gottfredson, 2015), this study includes a total of 46 structured items. The preliminary sample size was determined to be at least 690. Considering a 20% invalid response rate, the final sample size was set at a minimum of 862. This study collected a total of 1,007 valid questionnaires, meeting the minimum sample size requirement.

### 2.2 Measuring tools

#### 2.2.1 Demographic data

Designed by the researchers based on the research objectives and content, after reviewing the literature and group discussions, it includes 12 sociodemographic characteristics such as age, gender, professional title, education level, marital status, whether the individual is an only child, years of work experience, job position, weekly working hours, frequency of night shifts per month, self-perceived health status, and degree of liking for the nursing profession.

#### 2.2.2 Thriving at work scale

Nurses' thriving at work was measured using the Thriving at Work Scale developed by Porath et al. (2012) in 2012 and translated and revised into Chinese by Han and Liu (2020). The scale contains 10 items divided into **two** dimensions: vitality and learning. It uses a 5-point Likert scale, ranging from “strongly disagree” to “strongly agree,” scored from 1 to 5 respectively, with a total score ranging from 10 to 50. Higher scores indicate a higher level of work growth among participants. The Cronbach's alpha coefficient of the Chinese version of this scale is 0.961 (Sang, 2024). In this study, the Cronbach's alpha coefficient was 0.820. According to Nunnally, a Cronbach's alpha coefficient above 0.7 is considered a good reliability (Nunnally, 1978).

#### 2.2.3 Utrecht work engagement scale

Work engagement was measured using the Chinese version of the Utrecht Work Engagement Scale, originally developed by Schaufeli and Bakker (2003) and translated and revised by Zhang and Gan (2005). The scale includes three dimensions with a total of 17 items. Items numbered 1, 4, 8, 9, 15, and 17 correspond to the vigor dimension; items 2, 5, 7, 10, and 13 correspond to the dedication dimension; and items 3, 6, 11, 12, 14, and 16 correspond to the absorption dimension. The scale uses a 7-point Likert scoring method, ranging from “never” to “always,” scored 0 to 6 points respectively, with a total possible score of 0 to 102. Higher scores indicate higher levels of individual work engagement. Generally, an average total score ≤ 2 points is considered low work engagement, 2–4 points is moderate, and ≥ 4 points is high (Yang, 2022). The Cronbach's alpha coefficient of the Chinese version of this scale is 0.979 (Wu et al., 2026). In this study, the overall Cronbach's alpha coefficient of the scale was 0.961.

#### 2.2.4 General self-efficacy scale

Nurse self-efficacy was measured using the General Self-Efficacy Scale developed by Schwarzer and Renner (2000) and translated and revised into Chinese by Wang et al. (2001). The scale consists of 10 items and uses a 4-point Likert scoring method: 1 = completely incorrect, 2 = incorrect, 3 = correct, and 4 = completely correct. The total score ranges from 10 to 40 points, with 10–20 indicating a low level, 21–30 a moderate level, and 31–40 a high level of self-efficacy (Liu et al., 2011). Higher scores represent higher levels of nurse self-efficacy. The Cronbach's alpha coefficient of the Chinese version of this scale is 0.870 (Wang et al., 2001). In this study, the scale's Cronbach's alpha coefficient was 0.948.

#### 2.2.5 Leisure crafting scale

The Leisure Crafting Scale, developed by Petrou and Bakker (2016) and translated and revised by Morales-García et al. (2024), was used to assess individuals' abilities in planning their leisure time. The Chinese version of the Leisure Crafting Scale contains 9 items and uses a 5-point Likert scale, where 1 = strongly disagree, 2 = disagree, 3 = uncertain, 4 = agree, and 5 = strongly agree. The total score ranges from 9 to 45 points, with 9–22 indicating a low level, 23–29 a moderate level, and 30–45 a high level (Sheng et al., 2024). Higher scores indicate a higher level of leisure crafting and stronger leisure time planning ability. The Cronbach's alpha coefficient of the Chinese version of this scale is 0.953 (Guo et al., 2022). In this study, the Cronbach's alpha coefficient was 0.944.

### 2.3 Data analysis

Statistical analysis was performed using SPSS 26.0 and AMOS 24.0 software. Measurement data were expressed as mean ± standard deviation ($\bar{x}$ ± s), while count data were described using frequency and percentage. Pearson correlation analysis was used to examine the relationships among variables. In the structural model, the model fit was assessed using the Goodness of Fit Index (GFI, >0.900), Normed Fit Index (NFI, >0.900), Incremental Fit Index (IFI, >0.900), Comparative Fit Index (CFI, >0.900), Relative Fit Index (RFI, >0.900), Tucker–Lewis Index (TLI, >0.900), and Root Mean Square Error of Approximation (RMSEA, <0.080) (Hu and Bentler, 1999). AMOS 24.0 was employed to explore the impact pathways of thriving at work, self-efficacy, and leisure crafting on work engagement. The Bootstrap method was used to test the significance of mediation effects. A two-tailed *p*-value of less than 0.05 was considered statistically significant.

## 3 Results

### 3.1 Characteristics

A total of 1,055 questionnaires were distributed, and 1,007 valid questionnaires were collected, resulting in an effective response rate of 95.45%. The general information of the participants is shown in Table 1. The comparison of work engagement scores among nurses with different characteristics indicated that differences in age, marital status, years of work experience, frequency of night shifts per month, self-perceived health status, and degree of liking for the nursing profession were statistically significant (*P* < 0.05).

**Table 1:** Comparison of general information and work engagement scores among nurses with different characteristics (*n* = 1,007).

Item	Group	N	Frequency (%)	Work engagement scores ($\bar{x}$ ±s)	*t/F*	*P*
Gender	Male	122	12.1	4.18 ± 1.12	1.490^a^	0.136
	Female	885	87.9	4.02 ± 1.15		
Age	≤ 30	514	51.0	3.96 ± 1.16	3.719^b^	0.025
	31–40	417	41.4	4.09 ± 1.14		
	≥41	76	7.6	4.30 ± 1.07		
Professional title	Nurse	413	41.0	4.02 ± 1.14	0.929^b^	0.426
	Registered nurse	269	26.7	3.99 ± 1.20		
	Senior registered nurse	312	31.0	4.10 ± 1.12		
	Associate chief nurse and above	13	1.3	4.39 ± 0.89		
Educational background	Associate degree and below	38	3.8	3.93 ± 1.26	0.204^b^	0.815
	Bachelor's degree	959	95.2	4.04 ± 1.15		
	Master's degree and above	10	1.0	3.99 ± 0.99		
Marital status	Single	461	45.8	3.91 ± 1.14	3.419^b^	0.017
	Married without children	81	8.0	4.16 ± 1.05		
	Married with children	455	45.2	4.14 ± 1.16		
	Divorced/other	10	1.0	4.10 ± 1.26		
One-child family	Yes	307	30.5	4.13 ± 1.16	1.727^a^	0.084
	No	700	69.5	4.00 ± 1.14		
Years of work experience	≤ 5	418	41.5	3.96 ± 1.15	2.990^b^	0.030
	6–10	190	18.9	3.99 ± 1.19		
	11–15	291	28.9	4.09 ± 1.10		
	≥16	108	10.7	4.31 ± 1.16		
Position	None	901	89.5	4.02 ± 1.15	1.450^b^	0.235
	Precepting teacher or nursing team leader	89	8.8	4.20 ± 1.12		
	Deputy head nurse and above	17	1.7	4.28 ± 1.05		
Weekly working hours	≤ 40	311	30.9	4.08 ± 1.22	0.724^b^	0.485
	41–50	488	48.4	3.99 ± 1.09		
	≥51	208	20.7	4.09 ± 1.17		
Monthly night shift frequency (times)	0	229	22.7	4.10 ± 1.10	5.545^b^	0.004
	1–4	504	50.1	4.12 ± 1.14		
	5–8	274	27.2	3.84 ± 1.18		
Self-perceived health status	Very bad	24	2.4	3.31 ± 1.70	43.741^b^	<0.001
	Poor	139	13.8	3.45 ± 1.05		
	Fairly good	628	62.4	3.97 ± 1.08		
	Very good	216	21.4	4.69 ± 1.03		
Degree of liking for the nursing profession	Like very much	459	45.6	4.67 ± 0.97	134.692^b^	<0.001
	Like	509	50.5	3.58 ± 0.94		
	Dislike	34	3.4	2.46 ± 1.09		
	Dislike very much	5	0.5	3.11 ± 2.16		

^a^*t*-value.

^b^*F*-value.

### 3.2 Nurses' work engagement, thriving at work, self-efficacy, and leisure crafting scores

As shown in Table 2, the average scores for each variables were calculated based on the total and mean item scores.

**Table 2:** Average scores of each variable item (*n* = 1,007, $\bar{x}$ ± s).

Variable	Number of items	Range	Total score ($\bar{x}$ ±s)	Mean score of items ($\bar{x}$ ±s)
Work engagement	17	2–102	68.67 ± 19.51	4.04 ± 1.15
Vigor	6	0–36	22.64 ± 7.57	3.77 ± 1.26
Dedication	5	0–30	21.52 ± 5.76	4.30 ± 1.15
Absorption	6	1–36	24.50 ± 7.07	4.08 ± 1.18
Thriving at work	10	14–50	39.83 ± 5.78	3.98 ± 0.58
Self-efficacy	10	10–40	29.18 ± 6.68	2.92 ± 0.67
Leisure crafting	9	9–45	31.97 ± 7.56	3.55 ± 0.84

### 3.3 Measurement model

The measurement model includes two latent variables comprising a total of five observed variables. Validation was conducted, and the standard factor load values of each observed variable were all above 0.7 (Afthanorhan et al., 2020), as shown in Table 3. Further confirmatory factor analysis of the measurement model indicated that the average variance extracted (AVE) for each factor was greater than 0.5, and the construct reliability (C.R) values were all above 0.7 (Wu, 2010), demonstrating good convergent validity of the data (Table 3).

**Table 3:** Verification results of confirmatory factor analysis.

Latent variable	Item	Factor load (FL)	Average variance extracted (AVE)	Construct reliability (CR)	Discriminant validity
Work engagement	Y1	0.903	0.793	0.920	0.891
	Y2	0.973			
	Y3	0.885			
Thriving at work	M1	0.905	0.844	0.914	0.919
	M2	0.728

According to the Pearson correlation coefficients, work engagement is positively correlated with thriving at work, self-efficacy, and leisure crafting (*r* = 0.698, 0.714, 0.585, *P* < 0.001). Thriving at work is positively correlated with self-efficacy and leisure crafting (*r* = 0.521, 0.531, *P* < 0.001), and self-efficacy is positively correlated with leisure crafting (*r* = 0.491, *P* < 0.001), as shown in Table 4. Finally, a discriminant validity test was conducted on the measurement model (Table 3). Discriminant validity is primarily used to assess the degree of distinction between different constructs or measured variables, thereby ensuring the accuracy of the variables and enhancing the explanatory and predictive power of the model. The results indicate that the correlation coefficients between variables are all less than the square root of the AVE values, demonstrating that the model has good discriminant validity (Cheung et al., 2024).

**Table 4:** Pearson correlation analysis among variables (*r*).

Variables	Work engagement	Vigor	Dedication	Absorption	Thriving at work	Self-efficacy	Leisure crafting
Work engagement	1.000						
Vigor	0.963	1.000					
Dedication	0.948	0.877	1.000				
Absorption	0.956	0.874	0.865	1.000			
Thriving at work	0.698	0.634	0.760	0.629	1.000		
Self-efficacy	0.714	0.703	0.677	0.666	0.521	1.000	
Leisure crafting	0.585	0.583	0.579	0.519	0.531	0.491	1.000

All *P*-values are <0.001.

### 3.4 Structural equation model

**Figure 2:**
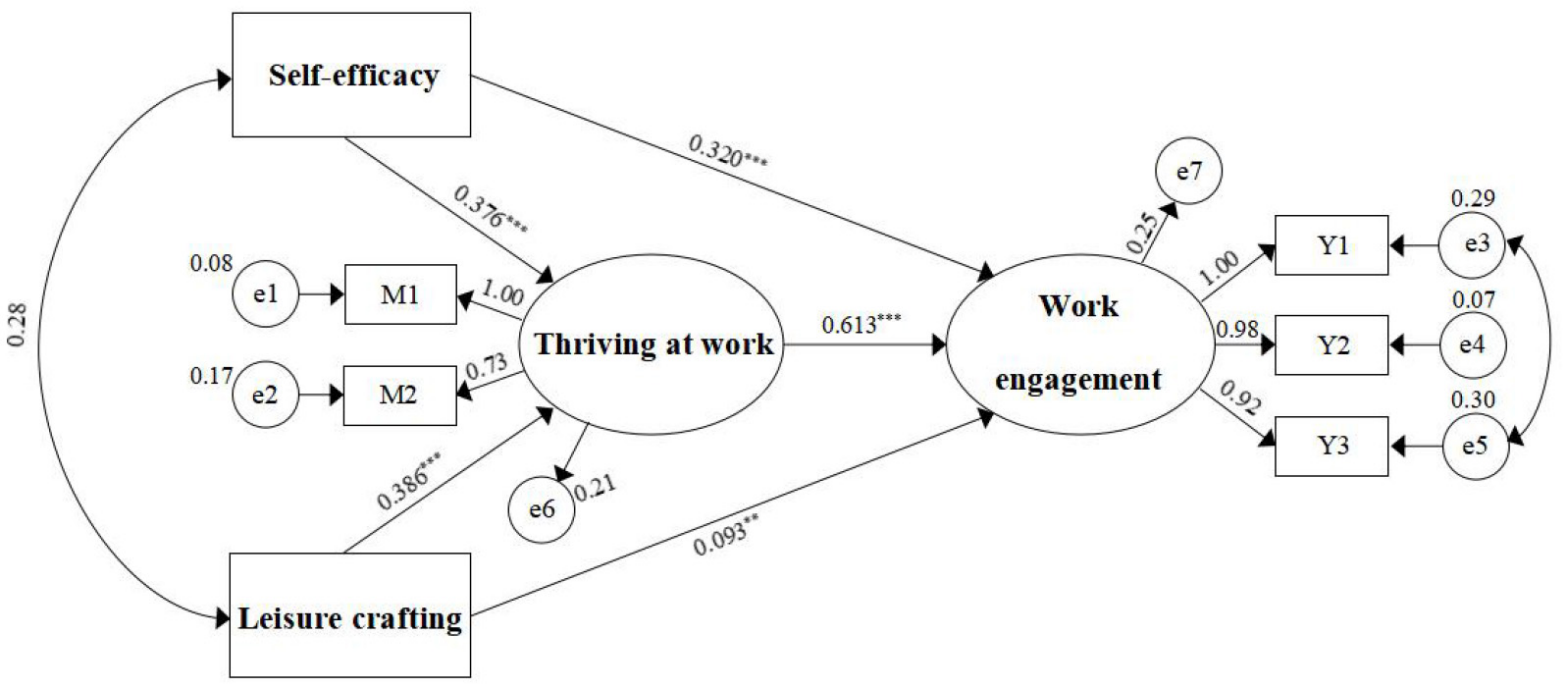
Structural Equation Model of Nurses' Work Engagement. M1, vitality; M2, learning; Y1, vigor; Y2, dedication; Y3, absorption; **P < 0.01, ***P < 0.001.

The theoretical framework of this study includes five paths. Based on this framework, a hypothetical model was constructed, and the model fit indices are fully presented in Table 5. According to the modification indices suggested by the software. Based on the initial model, we added the correlation between e3 and e5 to obtain the modified model. The fit of the modified model is better than that of the hypothetical model, with all hypothesized paths being significant (*P* < 0.05). Table 5 presents a comparison of the fit between the initial and modified models, where GFI, NFI, IFI, CFI, RFI, and TLI all meet the fit criteria. The RMSEA is slightly above the fit standard of 0.080, but still within an acceptable range. The final model, which includes a total of five paths, is shown in Figure 2. The parameter estimates among variables in the structural equation model of nurses' work engagement are shown in Table 6. Among them, the explained variance for work engagement is 80.5%, and for thriving at work, it is 43.3%.

**Table 5:** Te fit indexes of the hypothesized model and the modified model.

Item	GFI	NFI	IFI	CFI	RFI	TLI	RMSEA
Reference standards	>0.900	>0.900	>0.900	>0.900	>0.900	>0.900	<0.080
Hypothesized model	0.925	0.948	0.949	0.949	0.890	0.894	0.102
Modified model	0.943	0.959	0.960	0.960	0.904	0.907	0.091

**Table 6:** Parameter estimates of variables for the modified model.

Paths	B (*SE*)	β	*P*	SMC
Self-efficacy → Work Engagement	0.574 (0.041)	0.320	<0.001	0.805
Leisure crafting → Work Engagement	0.127 (0.032)	0.093	<0.001	
Thriving at work → Work Engagement	1.157 (0.067)	0.613	<0.001	
Self-efficacy → Thriving at work	0.340 (0.028)	0.376	<0.001	0.433
Leisure crafting → Thriving at work	0.278 (0.023)	0.386	<0.001	

S.E., Standard Error, β, Standardized estimate, SMC, Squared multiple correlation.

### 3.5 Effect

In this study, thriving at work had the greatest impact on the outcome variable of work engagement, while leisure crafting had the greatest impact on the mediating variable of thriving at work. All five hypothesized path effects in this study were statistically significant. Additionally, the indirect effects of self-efficacy on work engagement and leisure crafting were also statistically significant. For details, as detailed in Table 7.

**Table 7:** Standardized direct, indirect, and total effects.

Dependent variable	Independent variable	Direct effect	Indirect effect	Total effect
Work engagement	Self-efficacy	0.320^***^	0.231^***^	0.551^***^
	Leisure crafting	0.093^***^	0.237^***^	0.330^***^
	Thriving at work	0.613^***^	–	0.613^***^
Thriving at work	Self-efficacy	0.376^***^	–	0.376^***^
	Leisure crafting	0.386^***^	–	0.386^***^

^***^P < 0.001.

## 4 Discussion

### 4.1 General information of nurses and the current status of work engagement, self-efficacy, leisure crafting, and thriving at work

The results of this study show that: (1) The average score for nurse work engagement items was 4.04 ± 1.15, slightly higher than the findings of Liu et al. (2025) and Cao et al. (2025), indicating that the nurses in this study demonstrated a relatively high level of work engagement. The reason for this may be that tertiary hospitals have relatively sufficient financial support and training resources, enabling the implementation of a more comprehensive compensation system. Nurses can see clear career advancement opportunities, which gives their work engagement a stronger sense of purpose. (2) The total score for nurse self-efficacy was 29.18 ± 6.68, at a moderate level and slightly lower than the results reported by Yang et al. (2022), suggesting that there is room for improvement in nurses' sense of self-efficacy. This may be because tertiary hospital patients often have complex conditions and nursing tasks are heavy, with nurses frequently facing high workloads and high-risk duties, increasing work pressure and thereby weakening confidence in their own abilities. (3) The total score for nurses' leisure crafting was 31.97 ± 7.56, slightly lower than the results of Zhang (2025) but significantly higher than those of Sheng et al. (2024), placing it at a moderately high level. This indicates that the nurses in this study have good abilities in planning their leisure time. Research shows that nurses with higher levels of leisure time management tend to have greater work efficiency and engagement, providing patients with higher quality and more efficient nursing services, reflecting the nurses' personal professional qualities and development potential (Sheng et al., 2024). (4) The total score for nurses' thriving at work was (39.83 ± 5.78), significantly higher than the results reported by Ren et al. (2025), placing it at a moderately high level. This indicates that the nurses in this study demonstrated a relatively high level of thriving at work. The reasons for this may be as follows: first, this study excluded nursing staff who were in internship, standardized training, or further education stages. All participants were nurses employed by tertiary hospitals, possessing a positive work attitude and mature professional skills. Additionally, more than half of the nurses (58.5%) had six or more years of work experience, providing them with rich clinical nursing expertise. Furthermore, the vast majority of nurses (90.8%) earned a monthly income of 5,001 yuan or more. A stable work environment and income ensured a good quality of life for the nurses, which in turn enhanced their enthusiasm for clinical nursing work and their sense of professional identity.

The results of the *t*-tests and the one-way analysis of variance indicated that differences in nurse work engagement scores among groups based on age, marital status, years of work experience, monthly night shift frequency, self-perceived health status, and degree of liking for the nursing profession were statistically significant (*P* < 0.05). Nurses aged 41 and above and those with 16 or more years of work experience demonstrated higher levels of work engagement, which is consistent with previous research findings (Keykha et al., 2025; Alharbi and Alrwaitey, 2023). Multiple studies have found that nurses' work engagement levels increase with age and years of experience, with more senior nurses typically exhibiting higher work engagement (Keykha et al., 2025; Alharbi and Alrwaitey, 2023). The reasons for this include that older nurses are generally in a stable phase of work and family life, with their children mostly in adolescence, meaning they have more time to dedicate to work. Additionally, longer work experience usually corresponds to higher organizational commitment, accompanied by mature professional identity and rich clinical experience, which helps enhance nurses' confidence, professional competence, and sense of control over their work, thereby boosting work engagement (Keykha et al., 2025).

In this study, married nurses without children exhibited the highest levels of work engagement, which is generally consistent with most previous research (Alharbi and Alrwaitey, 2023; Zhong et al., 2025). Studies have shown that married nurses tend to have higher work engagement than unmarried nurses. Due to their multiple social and family roles, married nurses may actually experience enhanced work engagement, demonstrating greater vitality and dedication (Xu et al., 2024; Okada et al., 2019). Additionally, positive spillover effects from family, such as emotional support and a sense of responsibility, help boost nurses‘ work enthusiasm and sense of accomplishment (Xu et al., 2024; Okada et al., 2019). However, a few studies have found that married nurses might have limited time and energy for work due to increased family burdens, leading to decreased work engagement (Zhong et al., 2025; Kurniasih et al., 2025). Therefore, the impact of marital status on nurses' work engagement needs to be considered in conjunction with factors such as age, years of work experience, and family support.

The research results show that nurses who work 5–8 night shifts per month, perceive their health status as very poor, and dislike the nursing profession have the lowest levels of work engagement. This may be because an increased frequency of night shifts significantly affects nurses' sleep quality, mental health, and job performance, leading to higher occupational stress and anxiety levels, as well as worsened sleep disorders, thereby reducing work engagement (Li and Tse, 2025; Li et al., 2022). Previous studies have indicated that frequent night shifts are closely associated with chronic fatigue, decreased attention, cognitive decline, burnout, and lower job satisfaction (Li et al., 2023; Hutapea and Hutapea, 2025). These findings suggest that nursing managers should fully consider the negative impact of night shift frequency on nurses' physical and mental health and optimize scheduling patterns by implementing flexible shifts and reducing consecutive night shifts to alleviate occupational stress and improve nurses‘ work engagement. Multiple studies have shown that the better nurses rate their own health, the higher their level of work engagement (Malagon-Aguilera et al., 2019; Ge et al., 2021). Nurses who perceive their health as very poor may struggle to handle high-intensity work tasks due to declining physical function. When faced with complex and ever-changing clinical situations, they often feel overwhelmed, which in turn affects their work engagement (Ge et al., 2021). Nursing managers should pay attention to nurses' health status by organizing regular health check-ups and conducting health lectures to offer necessary health support and assistance. Additionally, nurses who do not like the nursing profession and lack a sense of identification and enthusiasm for nursing work are prone to negative emotions and find it difficult to fully commit themselves to their duties (Zhong et al., 2025). Nursing managers should strengthen professional guidance and psychological counseling for nurses to help them establish correct professional values and perspectives, thereby stimulating their work enthusiasm and motivation.

### 4.2 Fit of the structural equation model for nurse work engagement

This study is theoretically supported by the COR Theory. After verification and modification of the structural model, the goodness-of-fit of the structural equation model for nurses' work engagement reached a satisfactory level. Most of the fit indices for the structural equation model of nurse work engagement performed well, but the RMSEA value was slightly high. Possible reasons include sample size limitations, heterogeneity of survey subjects, complexity of path specifications, and measurement errors in cross-sectional study data (Shi et al., 2019). The critical values of fit indices for structural equation models should be judged comprehensively based on model complexity and sample size, rather than mechanically applying a single standard (Wen et al., 2004). Moreover, this study's theoretical model is well-supported by literature and has logically sound research hypotheses. Therefore, the model is still considered to have explanatory power, and the results are of reference value.

Independent variables such as self-efficacy, leisure crafting, and thriving at work can directly influence work engagement. These variables explain 80.5% of the variance in work engagement, which is higher than the 57.0% explained by job crafting on nurse work engagement reported by Baghdadi et al. (2021). The reason for this may be that the variables in this study cover aspects such as personal ability perception, external work resources, and career development, providing a more comprehensive explanation of work engagement. Additionally, nurses in tertiary hospitals place great importance on personal work growth; in this study, nurses' thriving at work was at a moderately high level. As thriving at work increases, the enrichment of resources and experiences has a significant positive effect on work engagement. Furthermore, self-efficacy and leisure crafting explain 43.3% of the variance in thriving at work.

### 4.3 The relationships between variables in the structural equation model of work engagement of nurses

The results of this study show that the higher a nurse's sense of self-efficacy, the greater their level of work engagement, which is consistent with previous research findings. When facing high-intensity work, self-efficacy positively predicts positive states such as work engagement (Van Wingerden et al., 2017). At the same time, the model verified that self-efficacy indirectly enhances nurses' work engagement by boosting thriving at work. This suggests that nurses with a high sense of self-efficacy are more confident in coping with challenges and difficulties at work. This positive psychological resource enables them to experience a higher level of thriving at work, thereby becoming more proactive and focused in their work tasks. This suggests that nursing managers should actively take measures to enhance nurses' self-efficacy in the workplace. By breaking down work goals, leveraging individual strengths to achieve these goals, helping nurses experience success, and providing timely, positive, and effective work feedback, nurses can receive constructive information that boosts their confidence and helps strengthen their self-efficacy. At the same time, attention should be given to improving nurses‘ professional identity, optimizing the work environment, and encouraging nurses to pursue continuing education, all of which are effective ways to enhance nurses' self-efficacy.

Leisure crafting (such as proactive growth and interest development during leisure time) refers to self-development activities carried out outside of work hours, requiring individuals to demonstrate greater positivity and initiative. Positive extrawork growth helps enhance nurses' professional reflection abilities and stress-coping skills (Pan et al., 2021), and is considered an important way to improve their work engagement, well-being, and professional flourishing (Pan et al., 2021). Similarly, research by Shi et al. (2021) shows that leisure crafting is a key factor in nurses' innovation activities, positively influencing innovative behaviors and promoting work growth and engagement. It is worth noting that leisure crafting not only directly enhances nurses' work engagement but also indirectly exerts an effect by boosting thriving at work. This suggests that nurses' proactive personal growth and interest development during non-working hours help accumulate psychological resources, improve professional skills, reflective abilities, and stress coping techniques, thereby achieving a higher level of thriving at work, ultimately promoting increased work engagement. This supports the core viewpoint of the COR theory—that individuals can generate positive occupational behavioral outcomes by actively acquiring and accumulating resources. Therefore, nursing managers should focus on cultivating nurses' leisure crafting abilities and actively encourage team nurses to proactively meet their individual psychological needs during off-hours (such as relaxation, growth, hobbies, etc.). Developing nurses' leisure crafting abilities emphasizes goal setting and reflection, which can be supported through offline training, personalized leisure plans, online platforms or apps, and other formats. Setting specific, measurable goals, tracking progress, and engaging in regular reflection can promote continuous improvement and enhance self-shaping abilities at the task, relational, and cognitive levels. This approach helps nurses proactively develop skills, relieve stress, and foster personal growth during their leisure time.

In this study, the path effect from thriving at work to work engagement was statistically significant, consistent with previous research. The findings indicate that nurses' thriving at work has a significant positive predictive effect on work engagement. Nurses with higher levels of thriving at work tend to exhibit higher levels of work engagement. Those with greater thriving at work demonstrate more energy, enthusiasm, and motivation for development, often showing higher work engagement and organizational commitment. These factors are considered key psychological and behavioral variables for improving nursing quality, reducing turnover, and promoting professional well-being (Zhai et al., 2023; Sharaf, 2021; Moloney et al., 2020). This suggests that nursing managers should pay attention to nurses' thriving at work and actively take measures to enhance it, such as providing proper guidance on nursing professional values, fostering a positive work environment, and ensuring reliable support from colleagues and leaders. Additionally, nursing managers should help staff identify, develop, and apply their strengths, improve self-efficacy and proactive growth willingness, and promote nurses' self-directed learning and career development.

### 4.4 Limitations

This study has certain limitations. First, the representativeness and diversity of the sample need to be improved. The sample in this study was drawn from a group of nurses at a tertiary hospital in Shaanxi Province, China. It is recommended that future research conduct multi-center studies to expand the types and scope of samples, thereby enhancing the generalizability and persuasiveness of the findings. Second, due to the limitations of cross-sectional survey research in establishing temporal and causal relationships, the inferences about the relationships between variables in this study rely on theory and previous research. It is suggested that future studies conduct interventional or qualitative research on factors influencing nurses' work engagement to further explore and predict these influencing factors.

## 5 Conclusion

This study based on the COR theory and previous research findings, constructed a structural equation model of nurse work engagement. The model explained 80.5% of the total variance in nurse work engagement and 43.3% of the total variance in thriving at work, analyzing the effect sizes of various variables on work engagement as well as the mediating role of thriving at work.

## 6 Implications for nursing management

Nurse work engagement is a crucial factor in ensuring the quality of nursing care, enhancing patient satisfaction, and promoting the development of the nursing profession. This study offers new insights for nursing managers to implement relevant measures to improve nurse work engagement. Considering the impact of self-efficacy, leisure crafting, and thriving at work on work engagement, nursing managers should pay attention to these factors and provide targeted interventions. First, nursing managers should prioritize the status of nurses' work engagement, as it not only directly affects nurses' job performance but also indirectly influences the quality of patient care and patient satisfaction. Second, managers should adopt corresponding intervention measures based on the variables affecting nurse work engagement. For example, addressing nurses' needs for thriving at work by offering training and promotion opportunities can foster their professional development. Additionally, enhancing nurses' self-efficacy and leisure crafting abilities, as well as optimizing job design to reduce burnout, can comprehensively improve nurses' work engagement and the quality of nursing services.

## Data Availability

The original contributions presented in the study are included in the article/supplementary material, further inquiries can be directed to the corresponding author.
